# Distant entanglement via photon hopping in a coupled cavity magnomechanical system

**DOI:** 10.1038/s41598-023-48825-8

**Published:** 2023-12-09

**Authors:** Amjad Sohail, Jia-Xin Peng, Abdelkader Hidki, Mohammad Khalid, S. K. Singh

**Affiliations:** 1 Department of Physics, Government College University, Allama Iqbal Road, Faisalabad, 38000 Pakistan; 2 State Key Laboratory of Precision Spectroscopy, Quantum Institute for Light and Atoms, Department of Physics, East China Normal University, Shanghai, 200062 China; 3 LPTHE, Department of Physics, Faculty of Sciences, Ibn Zohr University, Agadir, Morocco; 4 Sunway Centre for Electrochemical Energy and Sustainable Technology (SCEEST), School of Engineering and Technology, Sunway University, No. 5 Jalan Universiti, Bandar Sunway, 47500 Petaling Jaya, Selangor Malaysia; 5 Manipal Institute of Technology, Manipal Academy of Higher Education, Manipal, Karnataka 576104 India; 6 Uttaranchal University, Dehradun, Uttarakhand 248007 India; 7 Graphene and Advanced 2D Materials Research Group (GAMRG), School of Engineering and Technology, Sunway University, Petaling Jaya, Selangor Malaysia

**Keywords:** Optical physics, Quantum physics

## Abstract

We theoretically propose a scheme to generate distant bipartite entanglement between various subsystems in coupled magnomechanical systems where both the microwave cavities are coupled through single photon hopping coupling strength Γ. Each cavity contains a magnon mode and phonon mode and this gives six excitation modes in our model Hamiltonian which are cavity-1 photons, cavity-2 photons, magnon and phonon in cavity-1, and magnon and phonon in cavity-2. We found that significant bipartite entanglement exists between indirectly coupled subsystems in coupled microwave cavities for an appropriate set of parameters regime. Moreover, we also obtain suitable cavity and magnon detuning parameters for a significant distant bipartite entanglement in different bipartitions. In addition, it can be seen that a single photon hopping parameter significantly affects both the degree as well as the transfer of quantum entanglement between various bipartitions. Hence, our present study related to coupled microwave cavity magnomechanical configuration will open new perspectives in coherent control of various quantum correlations including quantum state transfer among macroscopic quantum systems.

## Introduction

Quantum entanglement is a fundamental property of quantum mechanics and has proven to be a key ingredient in various quantum technologies as well as it is an important area of study in both theoretical and experimental quantum physics^1^. In continuous variables (CV) quantum systems which are described by Gaussian states, a very well known mathematical formulation to quantify the amount of bipartite entanglement present is the logarithmic negativity^2^. In the early stages of quantum technology, seminal theoretical and experimental investigations mainly explored only microscopic systems, such as atoms, trapped ions, etc to obtain quantum entanglement^3^. However, the realization of quantum entanglement for various quantum protocols in practical applications and larger-scale quantum technologies often necessitates working at macroscopic level. Major advancements in nanotechnology already provided novel platform such as cavity optomechanical system to study macroscopic bipartite entanglement between a single cavity mode and a vibrating mirror^2^. Subsequently, several studies such as entanglement of two vibrating mirrors^4–8^ , Entanglement of multiple cavity modes coupled to single vibrating mirror^9–11^, entanglement in Laguerre-Gaussian cavity system^12–18^ explored macroscopic quantum entanglement in cavity optomechanical systems.

Ferrimagnetic materials such as yttrium iron garnet (YIG) sphere based cavity magnomechanical systems also offer a robust platform for studying the macroscopic quantum phenomena^19–22^. In cavity magnomechanical systems, YIG sphere is the most favourable ferrimagnetic materials due to its extremely high spin density and low decay rates of collective spin excitations known as Kittel mode^23,24^. This leads to the strong coupling of Kittel mode with the microwave cavity photons which leads to the vacuum Rabi splitting and cavity-magnon polaritons. So, these systems also provides a promising platform for the study of strong interactions between light and matter^25–27^. Moreover, many others interesting quantum phenomena such as magnon induced transparency^28–31^, coherent feedback enhanced entangelemnt^32,33^, magnon dark modes^34^, bistability^35,36^, magnon Kerr effect^37–41^ , microwave-optical conversion^42^, and magnon blockade^43,44^, successfully explored in cavity magnomechanical systems. In 2018, J. Lie et. al first studied the magnon-photon-phonon entanglement in cavity magnomechanical systems^27^. Many other theoretical works such as entanglement between two microwave fields^45,46^, entanglement between two YIG spheres^47^, Kerr enhanced entanglement between two magnon modes^48,49^, enhancement of photon-magnon entanglement using optical parametric amplifier^50,51^ and magnon squeezing effects^52,53^ were proposed to enhance macroscopic quantum entanglement as well as remote magnon-magnon entanglement between two massive ferrimagnetic spheres^54,55^.

Distant entanglement, also well known as long-distance entanglement, refers to the phenomenon to generate and maintain bipartite entanglement between quantum systems that are physically separated by finite distances^56^. The study of distant entanglement is an active area of research due to its significant applications in quantum information science, quantum communication and quantum networks. Recently, only few theoretical works studied the perfect transfer of bipartite entanglement and quantum steering between different subsystems in coupled cavity magnomechanical system where both the microwave cavities are coupled through single photon hopping factor^57–60^. Motivated by these works, we theoretically investigate coupled magnomechanical system to generate distant bipartite entanglement between different bi-partitions. Therefore, we emphasis on the possibility of generation of distant entanglements via single photon hoping. Furthermore, such a well-designed coupled magnomechanical system can be utilized to entangle and then the transfer of entanglement between different distant bosonic modes.

This paper is organized as follows: In Sect. "The model", we introduce theoretical model Hamiltonian whereas in Sect. "Quantum dynamics and entanglement of the coupled magnomechanical system", we evaluate quantum Langevin equations (QLEs) and also discuss in details about mathematical formulation of bipartite entanglement between different bipartitons. Numerical Results and related discussions are given in details in Sect. "Results and discussion", whereas we conclude our results in Sect. "Conclusion".

## The model

**Figure 1:**
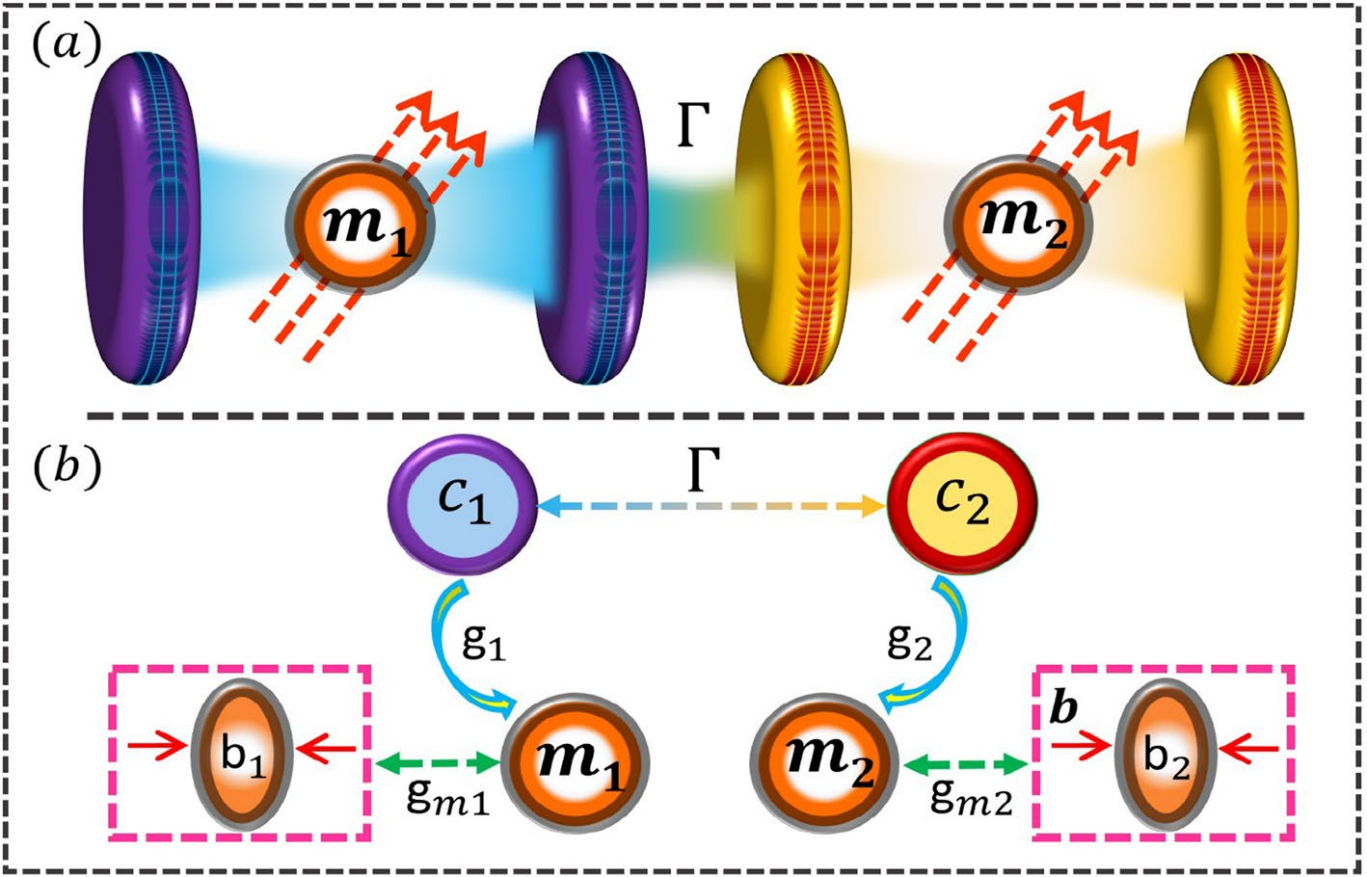
(**a**) Graphical representation of the coupled cavity magnomechanical system. Each MV cavity contain a magnon mode in a YIG sphere couples that interact with the cavity mode via magnetic dipole interaction. Furthermore, magnon mode interact with phonon mode via magnetostrictive interaction. The magnetic field of the each cavity modes is set to be in the x-direction, while the drive magnetic field (bias magnetic field) is considered along y-direction (z-direction). (**b**) The linear coupling diagram of each cavity magnomechanical system is shown. The two cavity modes are coupled via photon hoping $\Gamma$, while a cavity mode photon $c_{1}$ ($c_{2}$) is coupled to the magnon mode $m_{1}$ ($m_{2}$), with coupling strength $g_{1}$ ($g_{2}$), which then coupled to a phonon mode $b_{1}$ ($b_{2}$) to with magnomechanical coupling strength $g_{m1}$ ($g_{m2}$).

The magnomechanical system under consideration consists of two MW cavities connected through single photon hoping factor $\Gamma$. As shown in Fig. 1, each cavity contain a magnon mode *m* and a phonon mode *b*. The magnons in the YIG sphere are considered to be quasiparticles which are integrated by a large-scale collective excitation of spins inside a ferrimagnet, e.g. a YIG sphere^61^. The coupling between the magnon and the MW-field is due to magnetic dipole interaction. In addition, the positioning of YIG sphere in each cavity field is in the zone where there is a maximum magnetic field (See Fig. 1). At the YIG sphere site, the magnetic field of the cavity mode is along the x axis while the drive magnetic field is along the y direction). Furthermore, the bias magnetic field is set in the z direction. In addition, the magnon and phonon modes are coupled to each other via magnetostrictive force, which yields the magnon-phonon coupling^62,63^. The resonance frequencies of the magnon and phonon modes affect the magnetostrictive interaction^24^. In the current study, the mechanical frequency is considered to be much smaller than the magnon frequency, which surely helps to set up the strong dispersive phonon-magnon interaction^61,64^. The Hamiltonian of the coupled magnomechanical system takes the form:

1 $$\begin{aligned} H/\hslash= & {} H_{0}+H_{int}+H_{d}, \end{aligned}$$

where

2 $$\begin{aligned} H_{0}= & {} \sum _{k=1}^{2}\left[ \omega _{k}c_{k}^{\dag }c_{k}+\omega _{m_{k}}m_{k}^{\dag }m_{k}+\frac{\omega _{b_{k}}}{2}\left( q_{k}^{2}+p_{k}^{2}\right) \right] , \end{aligned}$$

3 $$\begin{aligned} H_{int}= & {} \sum _{k=1}^{2}\left[ g_{k}\left( c_{k}m^{\dag }+c_{k}^{\dag }m\right) +g_{mk}m_k^{\dag }m_{k}q_{k}\right] +\Gamma \left( c_{1}c_{2}^{\dag }+c_{1}^{\dag }c_{2}\right) , \end{aligned}$$

4 $$\begin{aligned} H_{d}= & {} i\Omega \sum _{k=1}^{2}\left[ m_{k}^{\dag }e^{-i\omega _{0k}t}-m_{k}e^{i\omega _{0k}t}\right] , \end{aligned}$$

where $c_{k}\left( c_{k}^{\dag }\right)$ and $m_{k}\left( m_{k}^{\dag }\right)$ are the annihilation (creation) operator of the the *k* cavity and magnon mode, respectively. Furthermore, $q_{k}$ and $p_{k}$ are the position and momentum quadratures of the respective mechanical mode of the magnon. In addition $\omega _{k}$, $\omega _{m_k}$, and $\omega _{b_k}$ denote the resonant frequencies of the *k*-th cavity mode, magnon mode, and mechanical mode. The magnon frequency $\omega _{m_k}$ can be finely tuned by adjusting the bias magnetic field *B* through the relation $\omega _{m_k} = \gamma _{0}B$, where $\gamma _{0}$ represents the gyromagnetic ratio. Furthermore, the optomagnonical coupling strength is given by

5 $$\begin{aligned} g_{k}= & {} \mathscr {V}\frac{c}{n_{r}}\sqrt{\frac{2}{\rho _{spin}V_{YS}}}, \end{aligned}$$

where $\mathcal {V}$ represents the Verdet constant of the YIG sphere, $\rho _{spin}$ represents the spin density, $n_{r}$ stands for the refractive index, and $V_{YS}=\frac{4\pi r^{3}}{3}$ corresponds to the volume of the YIG sphere^65^. We examined the scenario of strong coupling, wherein the interaction between the *k*-th cavity mode and magnon mode $g_{k}$ surpasses the decay rates of both the magnon and the cavity modes, i.e. $g_{k}>\kappa _{m_k}$, $\kappa _{k}$^25,65,66^. Furthermore, $g_{mk}$ denotes the interaction strength between magnons and phonons, which is generally considered to be quite small. However, it can be improved by employing a MW field to drive the YIG sphere. The Rabi frequency $\Omega =( \sqrt{5}/4) \gamma _{0}\sqrt{N_{spin}}B_{0}$^67,68^ denotes the coupling strength of the drive field with frequency $\omega _{0}$ and amplitude $B_{0}=3.9\times 10^{-9}$T, where $N_{spin}=\rho V_{YS}$ is the total number of spins with the spin density of the YIG $\rho _{spin} =4.22\times 10^{27}m^{-3}$ and $\gamma _{0}=28$GHz/T. It is also crucial to emphasize that the collective motion of the spins is reduced to bosonic operators *m* and $m^{\dagger }$ through the Holstein-Primakoff transformation. Additionally, the Rabi frequency $\Omega$ is obtained based on the fundamental assumption of having low-lying excitations, specifically when $2Ns\gg \langle m^{\dagger }m \rangle$, where $s=\frac{5}{2}$ represents the spin value of the Fe$^{3+}$ ion in the ground state of YIG. Moreover, $\Gamma$ represents the single photon hopping strength between the two cavity mode which is mainly controlled by adjusting the distance between two microwave cavities. However, there are other factor which affect the photon hopping strength like cavity detuning, cavity decay as well as transmission and reflection of the cavity mirrors.

The Hamiltonian of the system can be written as following under the rotating wave approximation at the drive frequency $\omega _0$:

6 $$\begin{aligned} H/\hslash= & {} \sum _{k=1}^{2}\left[ \Delta _{k}c_{k}^{\dag }c_{k}+\Delta _{m_{k}}m_{k}^{\dag }m_{k}+\frac{\omega _{b_{k}}}{2}\left( q_{k}^{2}+p_{k}^{2}\right) +g_{mk}m_{k}^{\dag }m_{k}q_{k} +g_{k}\left( c_{k}m_{k}^{\dag } +c_{k}^{\dag }m_{k}\right) +i\Omega \left( m_{k}^{\dag }-m_{k}\right) \right] \nonumber \\{} & {} +\Gamma (c_{1}c_{2}^{\dag }+c_{1}^{\dag }c_{2}), \end{aligned}$$

where $$\Delta _{k}=\omega _{k}-\omega _{0k}$$ and $$\Delta _{m_{k}}=\omega _{m_{k}}-\omega _{0k}$$.

## Quantum dynamics and entanglement of the coupled magnomechanical system

Because of the interaction between the magnomechanical system and its environment, the system will experience influences from cavity decay, magnon damping, and mechanical damping. By considering these dissipative factors, the system’s dynamics can be characterized by a set of quantum Langevin equations :

7 $$\begin{aligned} \dot{q}_{k}= & {} \omega _{b_{k}}p_{k}, \end{aligned}$$

8 $$\begin{aligned} \dot{p}_{k}= & {} -\omega _{b}q_{k}-\gamma _{b}p_{k}-g_{mk}m^{\dag }_{k}m_{k}+\xi _{k}, \end{aligned}$$

9 $$\begin{aligned} \dot{c}_{k}= & {} -\left( i\Delta _{k}+\kappa _{k}\right) c_{k}-ig_{k}m_{k}+\Gamma c_{j}+ \sqrt{2\kappa _{a}}c_{k}^{in}, (j=1,2, j \ne k) \end{aligned}$$

10 $$\begin{aligned} \dot{m}_{k}= & {} -\left( i\Delta _{m_{k0}}+\kappa _{m}\right) m_{k}-ig_{k}c_{k}-ig_{mk}m_{k}q_{k}+\Omega _{k} + \sqrt{2\kappa _{m_k}}m_k^{in}, \end{aligned}$$

where $\kappa _{k}$($\kappa _{m_k}$) represents the decay rate of the *k*-th cavity (magnon) mode and $\gamma _{b}$ is the damping rate of the *k*-th mechanical mode. $\xi$, $m^{in}$, and $c_{k}^{in}$ are operators for input noise associated with the *k*-th mechanical, magnon, and cavity modes, respectively. These noise operators are defined by the following correlation functions^69^:

11 $$\begin{aligned} \left\langle \xi _k (t)\xi _k (t^{\prime })\right\rangle +\left\langle \xi _k (t^{\prime })\xi _k (t)\right\rangle /2= & {} \gamma _{b} [2n_{b_k}(\omega _{b_k})+1]\delta (t-t^{\prime }), \end{aligned}$$

12 $$\begin{aligned} \left\langle m_k^{in\dag }(t)m_k^{in}(t^{\prime })\right\rangle= & {} n_{m_k}(\omega _{m_k})\delta (t-t^{\prime }), \end{aligned}$$

13 $$\begin{aligned} \left\langle m_k^{in}(t)m_k^{in\dag }(t^{\prime })\right\rangle= & {} [n_{m_k}(\omega _{m_k})+1]\delta (t-t^{\prime }), \end{aligned}$$

14 $$\begin{aligned} \left\langle c_{k}^{in}(t)c_{k}^{in\dag }(t^{\prime })\right\rangle= & {} [n_{k}(\omega _{k})+1]\delta (t-t^{\prime }), \end{aligned}$$

15 $$\begin{aligned} \left\langle c_{k}^{in\dag }(t)c_{k}^{in}(t^{\prime })\right\rangle= & {} n_{k}(\omega _{k})\delta (t-t^{\prime }), \end{aligned}$$

The equilibrium mean number of thermal photons, magnons, and phonons is expressed as $n_{f}(\omega _{f})=[\exp (\frac{\hslash \omega _{f}}{k_{b}T} )-1]^{-1}$ for $f=c_k,m_k,b_k$. Here, $k_{b}$ represents the Boltzmann constant and *T* denotes the temperature of the environment.

If the magnon mode experiences strong excitation, it implies that $\left| \left\langle m\right\rangle \right| \gg 1$. Furthermore, the two microwave cavity fields exhibit large amplitudes due to interactions with the beam splitter interaction between the cavity magnon modes. This allows us to simplify the QLEs by expressing any operator as the sum of its mean value and its fluctuation, i.e. $o=\left\langle o\right\rangle +\delta o$^70–72^, where $(o=p_k,q_k,c_{k},m_k)$, and then substitute this into Eqs. (7), (8), (9) and (10). The mean values of the dynamic operators can be calculated as follows:

16 $$\begin{aligned} \left\langle p _{k}\right\rangle= & {} 0, \end{aligned}$$

17 $$\begin{aligned} \left\langle q _{k}\right\rangle= & {} \frac{-g_{mk}}{\omega _{b}}\left| \left\langle m _{k}\right\rangle \right| ^{2}, \end{aligned}$$

18 $$\begin{aligned} \left\langle m_{k}\right\rangle= & {} \frac{\Omega _{k}-ig _{k}\left\langle c _{k}\right\rangle }{i\Delta _{m _{k}}+\kappa _{m _{k}}}, \end{aligned}$$

19 $$\begin{aligned} \left\langle c_{1}\right\rangle= & {} \frac{ig_{1}\Omega _{1}\Lambda _{2}-\Gamma g_{2}\Omega _{2}(\kappa _{m_{1}}+i\Delta _{m_{1}})}{\alpha _{1}\alpha _{2}+\Gamma ^{2}\left( i\Delta _{m_{1}}+\kappa _{m_{1}}\right) \left( i\Delta _{m_{2}}+\kappa _{m_{2}}\right) }, \end{aligned}$$

20 $$\begin{aligned} \left\langle c_{2}\right\rangle= & {} \frac{ig_{2}\Omega _{2}\Lambda _{1}-\Gamma g_{1}\Omega _{1}(\kappa _{m_{2}}+i\Delta _{m_{2}})}{\alpha _{1}\alpha _{2}+\Gamma ^{2}\left( i\Delta _{m_{2}}+\kappa _{m_{2}}\right) \left( i\Delta _{m_{1}}+\kappa _{m_{1}}\right) }, \end{aligned}$$

where $\Lambda _{k}=(i\Delta _{k}+\kappa _{k})(i\Delta _{m_{k}}+\kappa _{m_{k}})+g_{k}^{2}$, $\Delta _{m_{k}}=\Delta _{m_{k0}}+g_{mk}\left\langle q_{k}\right\rangle$ represents the effective magnon mode detuning which includes the slight shift of frequency due to the magnetostrictive interaction.

Now, we introduce the quadrature for the linearised quantum Langevin equations describing fluctuations are: $\delta x=\frac{1}{\sqrt{2}}(\delta m-\delta m^{\dag })$, $\delta y=\frac{1}{\sqrt{2}i}(\delta m-\delta m^{\dag })$, $\delta X_{k}=\frac{1}{\sqrt{2}}(\delta c_{k}-\delta c_{k}^{\dag })$, $\delta Y_{k}=\frac{1}{\sqrt{2}i}(\delta c_{k}-\delta c_{k}^{\dag })$ can be written as

21 $$\begin{aligned} \dot{\mathscr {F}}(t)=\mathscr {M}\mathscr {F}(t)+\mathscr {N}(t), \end{aligned}$$

where $\mathscr {F}(t)$ and $\mathscr {N}(t)$ denote the vectors of quantum fluctuations and input noise, respectively. They are defined as:

$$\begin{aligned} \mathscr {F}(t)= & {} [\delta C_{XY}(t),\delta M_{xy}(t),\delta Q_{qp}(t)]^{T},\\ \mathscr {N}(t)= & {} [\mathscr {N}_{XY},\mathscr {N}_{xy},\mathscr {N}_{qp}]^{T}, \end{aligned}$$

where

$$\begin{aligned} \delta C_{XY}(t)= & {} \delta X_{1}(t),\delta Y_{1}(t),\delta X_{2}(t),\delta Y_{2}(t), \\ \delta M_{xy}(t)= & {} \delta x_{1}(t),\delta y_{1}(t),\delta x_{2}(t),\delta y_{2}(t), \\ \delta Q_{qp}(t)= & {} \delta q_{1}(t),\delta p_{1}(t),\delta q_{2}(t),\delta p_{2}(t), \end{aligned}$$

and

$$\begin{aligned} \mathscr {N}_{XY}= & {} \sqrt{2k_{1}}X_{1}^{in}(t),\sqrt{2k_{1}}Y_{1}^{in}(t), \sqrt{2k_{2}}X_{2}^{in}(t),\sqrt{2k_{2}}Y_{2}^{in}(t), \\ \mathscr {N}_{xy}= & {} \sqrt{2k_{m}}x_{1}^{in}(t),\sqrt{2k_{m}}y_{1}^{in}(t), \sqrt{2k_{m}}x_{2}^{in}(t),\sqrt{2k_{m}}y_{2}^{in}(t), \\ \mathscr {N}_{qp}= & {} 0,\xi _{1}(t),0,\xi _{2}(t). \end{aligned}$$

In addition, the drift matrix $\mathscr {M}$ of the present coupled magnomechanical system can be expressed as

22 $$\begin{aligned} \mathscr {M}=\left[ \begin{array}{cccccccccccc} -\kappa _{1} &{} \Delta _{1} &{} 0 &{} \Gamma &{} 0 &{} g_{1} &{} 0 &{} 0 &{} 0 &{} 0 &{} 0 &{} 0 \\ -\Delta _{1} &{} -\kappa _{1} &{} -\Gamma &{} 0 &{} -g_{1} &{} 0 &{} 0 &{} 0 &{} 0 &{} 0 &{} 0 &{} 0 \\ 0 &{} \Gamma &{} -\kappa _{2} &{} \Delta _{2} &{} 0 &{} 0 &{} 0 &{} g_{2} &{} 0 &{} 0 &{} 0 &{} 0 \\ -\Gamma &{} 0 &{} -\Delta _{2} &{} -\kappa _{2} &{} 0 &{} 0 &{} -g_{2} &{} 0 &{} 0 &{} 0 &{} 0 &{} 0 \\ 0 &{} g_{1} &{} 0 &{} 0 &{} -\kappa _{m_{1}} &{} \Delta _{m_{1}} &{} 0 &{} 0 &{} -G_{1} &{} 0 &{} 0 &{} 0 \\ -g_{1} &{} 0 &{} 0 &{} 0 &{} -\Delta _{m_{1}} &{} -\kappa _{m_{1}} &{} 0 &{} 0 &{} 0 &{} 0 &{} 0 &{} 0 \\ 0 &{} 0 &{} 0 &{} g_{2} &{} 0 &{} 0 &{} -\kappa _{m_{2}} &{} \Delta _{m_{2}} &{} 0 &{} 0 &{} -G_{2} &{} 0 \\ 0 &{} 0 &{} -g_{2} &{} 0 &{} 0 &{} 0 &{} -\Delta _{m_{2}} &{} -\kappa _{b_{2}} &{} 0 &{} 0 &{} 0 &{} 0 \\ 0 &{} 0 &{} 0 &{} 0 &{} 0 &{} 0 &{} 0 &{} 0 &{} 0 &{} \omega _{b_{1}} &{} 0 &{} 0 \\ 0 &{} 0 &{} 0 &{} 0 &{} 0 &{} G_{1} &{} 0 &{} 0 &{} -\omega _{b_{1}} &{} -\gamma _{b_{1}} &{} 0 &{} 0 \\ 0 &{} 0 &{} 0 &{} 0 &{} 0 &{} 0 &{} 0 &{} 0 &{} 0 &{} 0 &{} 0 &{} \omega _{b_{2}} \\ 0 &{} 0 &{} 0 &{} 0 &{} 0 &{} 0 &{} 0 &{} G_{2} &{} 0 &{} 0 &{} -\omega _{b_{2}} &{} -\gamma _{b_{2}} \end{array} \right] , \end{aligned}$$

where $G_{k}=i\sqrt{2}g_{mk}\left\langle m_{k} \right\rangle$ governs the effective magnomechanical coupling strength. Furthermore, using Eq. (4), By utilizing strong magnon drive, the effective magnomechanical coupling strength can be increased..

**Table 1:** Adopted notation for the different bipartite subsystem entanglement.

Bipartite subsystem	Entanglement symbol	Bipartite subsystem	Entanglement symbol
Cavity 1-cavity 2	$E_{c-c}^{N}$		
Cavity 1-magnon 2	$E^{N}_{c_{1}-m_{2}}$	Cavity 2-magnon 1	$E^{N}_{c_{2}-m_{1}}$
Cavity 1-phonon 2	$E^{N}_{c_{1}-b_{2}}$	Cavity 2-phonon 1	$E^{N}_{c_{2}-b_{1}}$

**Table 2:** Parameters used in our numerical simulations.

Parameters (symbol)	Value	Parameters (symbol)	Value
Cavity decay rates ($\kappa _{1}=\kappa _{2}=\kappa$)	$2\pi \times$ 1 MHz	Magnon decay rate ($\kappa _{m}$)	$2\pi \times$1 MHz
Phonon frequency ($\omega _{b}$)	$2\pi \times$10 MHz	Cavity frequency ($\omega _{a}$)	$2\pi \times$10 GHz
Mechanical damping rate ($\gamma _{b}$)	$2\pi \times$100 Hz	Optomagnonical couplings($g_{k}$)	$2\pi \times$3.2 MHz
Drive magnetic field (B)	$3.9\times 10^{-5}$T	Magnomechanical coupling($g_{mk}$)	$2\pi \times$0.3 Hz
Power ($\wp =\frac{B^{2}\pi r^{2} c}{2\mu ^{2}}$)	9.8 mW	Spin density ($\rho$)	$4.22\times 10^{27}m^{-3}$
YIG sphere diameter (D)	250 $\mu$m	Temperature (T)	10 mK

**Figure 2:**
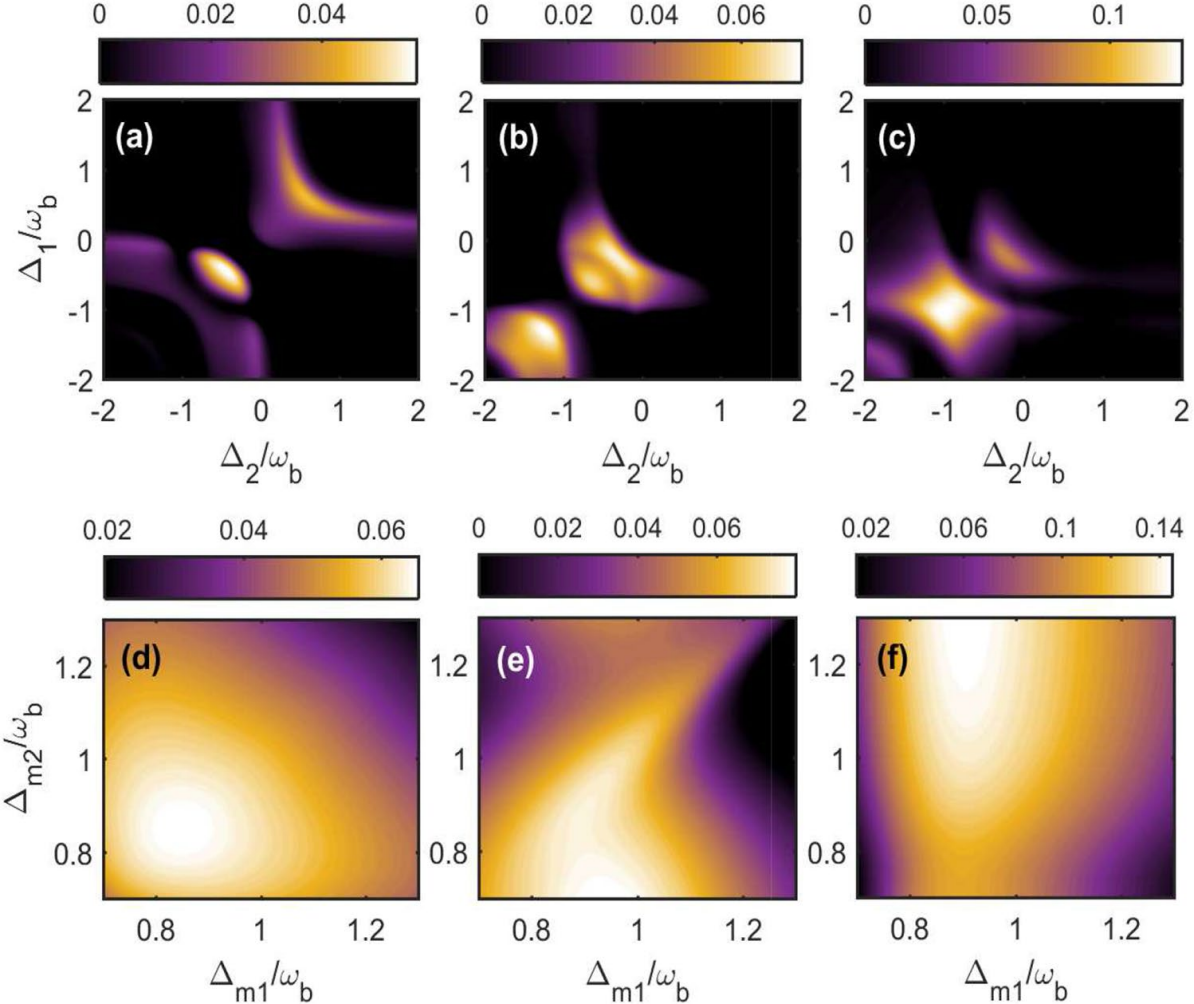
(Color online) Density plot of bipartite entanglement in (**a**,**d**) $E_{c_{1}-c_{2}}^{N}$; in (**b**,**e**) $E_{c_{1}-m_{2}}^{N}=E_{c_{2}-m_{1}}^{N}$ and in (**c**,**f**) $E_{c_{1}-b_{2}}^{N}=E_{c_{2}-b_{1}}^{N}$ versus cavity detunings $\Delta _{1}/\omega _{b}$ and $\Delta _{2}/\omega _{b}$ in (**a**–**c**) for $\Delta _{m_{1}}=\Delta _{m_{2}}=\omega _{b}$ whereas varying both magnon detunings $\Delta _{m_{1}}/\omega _{b}$ and $\Delta _{m_{2}}/\omega _{b}$ in (**d**–**f**) for both the cavity detunings fixed at $\Delta _{1} = \Delta _{2}=\omega _{b}$. The other parameters are given in Table 2.

Now we investigate the entanglement between various bipartite subsystems with an emphasis on the entanglements of the indirectly coupled mode. According to the Routh-Hurwitz criterion^73^, the system achieves stability only when we obtained negative real parts of all eigenvalues of the drift matrix $\mathcal {M}$, which is verified throughout this manuscript and therefore, the success of our approach hinges on the stability of the proposed system. The system under study is characterized by an $12\times 12$ covariance matrix *V* and its corresponding elements defined as:

23 $$\begin{aligned} V_{ij}(t)=\frac{1}{2}\left\langle \mathscr {F}_{i}(t)\mathscr {F} _{j}(t^{\prime })+\mathscr {F}_{j}(t^{\prime })\mathscr {F}_{i}(t)\right\rangle , \end{aligned}$$

The following Lyapunov equation can be utilized to determine the covariance matrix of our coupled magnomechanical system^74,75^

24 $$\begin{aligned} \mathscr {M}V+V\mathscr {M}^{T}=-\mathscr {D}, \end{aligned}$$

where $\mathscr {D}=$ diag$[\kappa _{1}\left( 2n_{1}+1\right) ,\kappa _{1}\left( 2n_{1}+1\right) , \kappa _{2}\left( 2n_{2}+1\right) ,\kappa _{2}\left( 2n_{2}+1\right) ,\kappa _{m_1}\left( 2n_{m_1}+1\right) ,\kappa _{m_1}(2n_{m_1}+1), \kappa _{m_2}\left( 2n_{m_2}+1\right) ,\kappa _{m_2}(2n_{m_2}+1), 0,\gamma _{b}\left( 2n_{b}+1\right) , 0,\gamma _{b}\left( 2n_{b}+1\right) ]$ represents the diffusion matrix, a diagonal matrix that characterizes noise correlations. Furthermore, Eq. (24) represents the steady-state correlation matrix. We use the logarithmic negativity to measures the degree of entanglement of the steady state, is given by^75–79^

25 $$\begin{aligned} E_{N}=\max [0,-\ln 2 \eta ^{-}], \end{aligned}$$

where $\eta ^{-}=$min eig$|\bigoplus ^{2}_{j=1}(-\sigma _{y})\widetilde{ \mathscr {V}_{4}}|$ represents the covariance matrix’s smallest symplectic eigenvalue. Here, $\widetilde{\mathscr {V}_{4}}=\varrho _{1|2}\mathscr {V} _{in}\varrho _{1|2}$, where $\mathscr {V}_{in}$ is a $4\times 4$ matrix obtained by extracting the relevant rows and columns from $\mathscr {V}_{4}$ for the chosen subsystems. The matrix $\varrho _{1|2}=\sigma _{z}\bigoplus 1$ =diag$(1,-1,1,1)$ performs partial transposition on covariance matrices. $\sigma$’s are the Pauli spin matrices in this context. Moreover, a positive value of logarithmic negativity, given as $E_{N}>0$, highlights the existence of bipartite entanglement between any two given modes in our cavity magnomechanical system.

## Results and discussion

As there are six different modes in this coupled cavity magnomechanical system, we investigate into details about the numerical results of different bipartite entanglements. So, we may get bipartite entanglement in any of two modes however the most significant part of our study is to explore the bipartite entanglement present in spatially distant subsystems which we have summarised in Table 1 with symbols. We have used the experimental feasible parameters in our study given as in Table 2.

**Figure 3:**
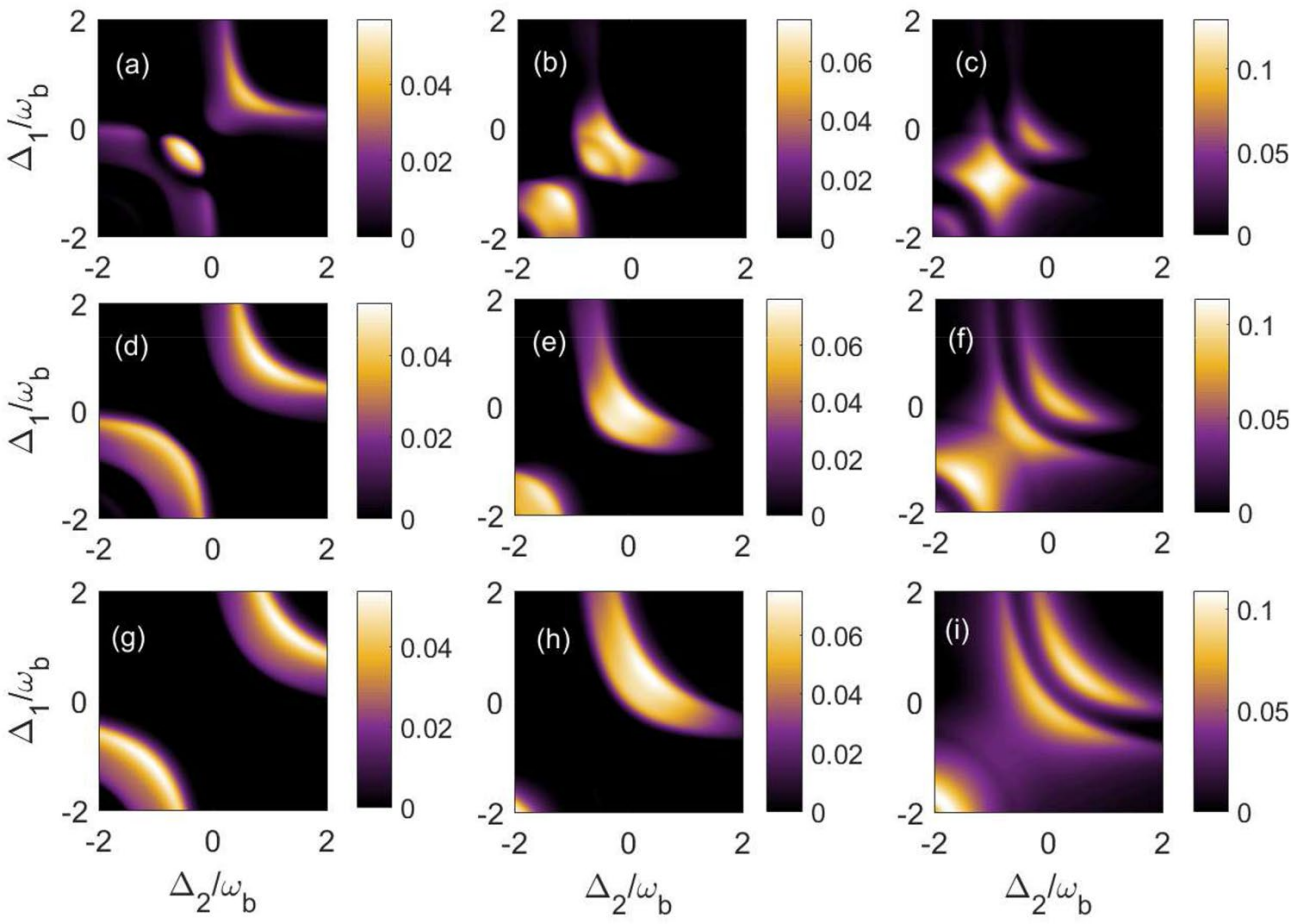
Density plot of bipartite entanglement in (**a**,**d**,**g**) $E_{c_{1}-c_{2}}^{N}$; in (**b**,**e**,**h**) $E_{c_{1}-m_{2}}^{N}=E_{c_{2}-m_{1}}^{N}$ and in (**c**,**f**,**i**) $E_{c_{1}-b_{2}}^{N}=E_{c_{2}-b_{1}}^{N}$ versus detunings $\Delta _{1}/\omega _{b}$ and $\Delta _{2}/\omega _{b}$. Here we have taken $\Gamma =0.5\omega _{b}$ for (**a**–**c**); $\Gamma =0.8\omega _{b}$ for (**d**–**f**) and $\Gamma =\omega _{b}$ for (**g**–**i**). We have fixed both the magnon detunings at $\Delta _{m_{1}}= \Delta _{m_{2}}=\omega _{b}$ The other parameters are given in Table 2.

In Fig. 2, we present five different distant bipartite entanglements as a function of the cavity detunings and gradually changing the hoping factor in our coupled magnomechanical system. For single photon hopping factor $\Gamma =0.5\kappa _{c}$ and keeping both the magnon detunings at blue sideband regime i.e. $\Delta _{m_{1}}=\Delta _{m_{2}}=\omega _{b}$, we obtain the optimal bipartite entanglements $E_{c_{1}-c_{2}}^{N}$ and $E_{c_{1}-m_{2}}^{N} \mathbf {(} E_{c_{2}-m_{1}}^{N}\text {)}$ at two different places, however the entanglement $E_{c_{1}-b_{2}}^{N} \mathbf {(} E_{c_{2}-b_{1}}^{N}\mathbf {)}$ is concentrated to a specific region of normalized cavity detunings as can be seen in Fig. 2a–c. It can be also seen that the optimal entanglement of these bipartitions exist for different cavity detunings which means that we can shift/transfer entanglement from one bipartition to another through gradually changing both the cavity detunings. Moreover, the bipartite entanglement between two cavity modes $E_{c_{1}-c_{2}}^{N}$ becomes maximum for $\Delta _{1}=\Delta _{2}= -0.5\omega _{b}$ although even if both the cavity detunings are resonant only with blue sideband regime i.e. $\Delta _{1}=\Delta _{2}= \omega _{b}$ we have significant amount of the bipartite entanglement in $E_{c_{1}-c_{2}}^{N}$ as shown in Fig. 2a. In Fig. 2b we study the bipartite entanglement $E_{c_{1}-m_{2}}^{N} \mathbf {(} E_{c_{2}-m_{1}}^{N}\text {)}$ which attains maximum value either when both the cavity detunings are resonant with the driving field, i.e. $\Delta _{1}=\Delta _{2}= 0$ or are resonant with red sideband regime, i.e. $\Delta _{1}=\Delta _{2}= -\omega _{b}$. Moreover when both the cavity detunings are kept in resonance (symetric case) with red sideband regime, the bipartite entanglement $E_{c_{1}-b_{2}}^{N} \mathbf {(}E_{c_{2}-b_{1}}^{N}\mathbf {)}$ attains its maximum value as shown in Fig. 2c. Furthermore, it can be seen that if cavity detunigs for both the cavities are kept fixed and in resonance with blue sideband regime i.e. $\Delta _{1}=\Delta _{2}= \omega _{b}$ then all the above mentioned bipartite entanglements have significant values on gradually varying $\Delta _{m_{1}}/\omega _{b}$ from 0.7 to 1.1 as shown in Fig. 2d–f, however, the entanglement can be transferred among these bipartitions by altering the $\Delta _{m_{2}}/\omega _{b}$. So, in this case to get significant amount of bipartite entanglements between different modes either both the cavity detunings or both the magnon detunings should be kept at blue sideband regime of the phonons due to deformation of YIG sphere. This is because it leads to anti-stokes process which results in significant cooling of phonons and enhance the bipartite entanglement between the different bipartions.

**Figure 4:**
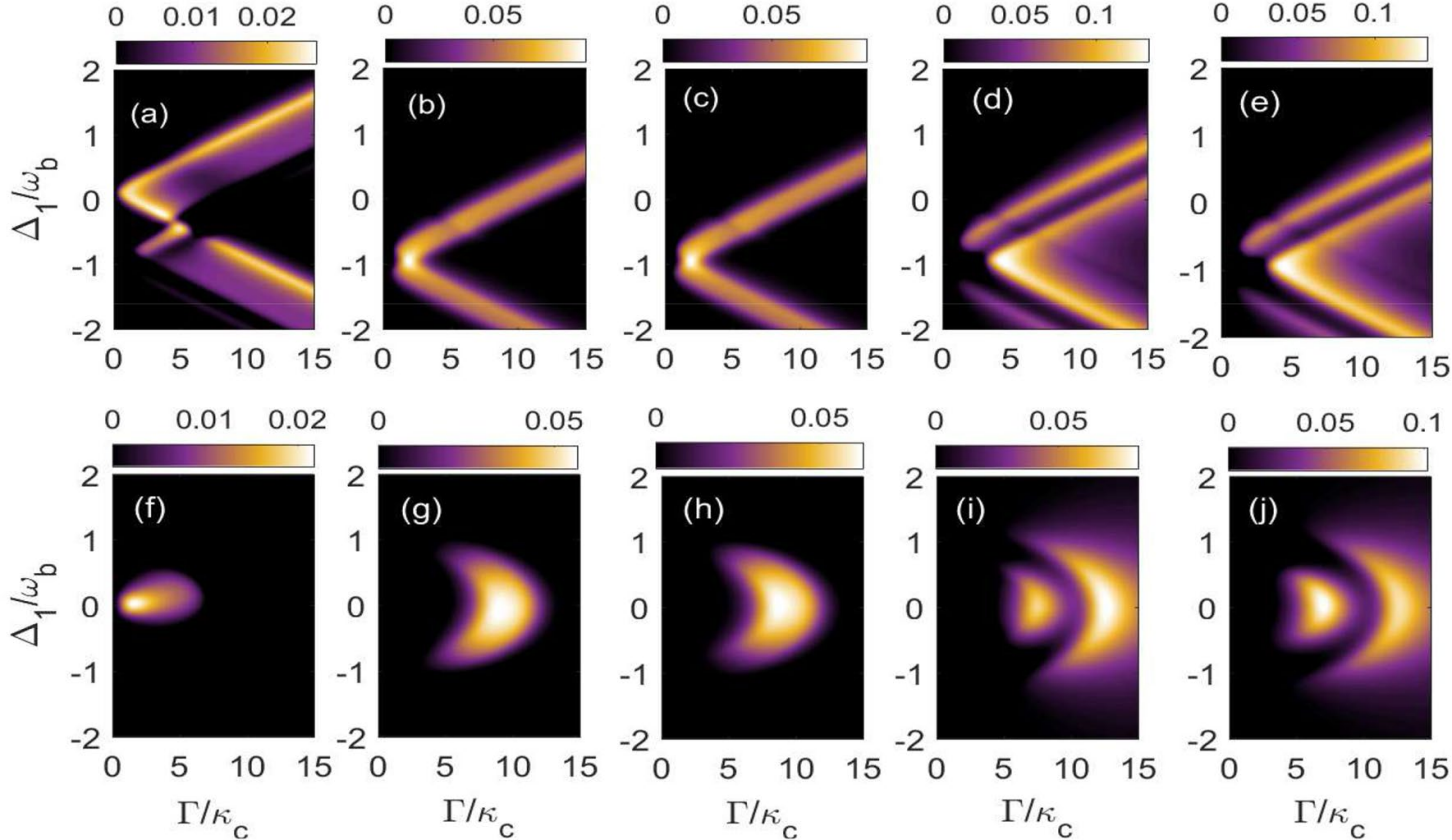
Density plot of bipartite entanglement in (**a**,**f**) $E^{N}_{c_{1}-c_{2}}$; in (**b**,**g**) $E^{N}_{c_{1}-m_{2}}$; in (**c**,**h**) $E^{N}_{c_{2}-m_{1}}$; in (**d**,**i**) $E^{N}_{c_{1}-b_{2}}$ and (**e**,**j**) $E^{N}_{c_{2}-b_{1}}$ versus $\Delta _{1}/\omega _{b}$ and $\Gamma / \omega _{b}$ for $\Delta _{2}=\omega _{b}$ in (**a**–**e**) and for $\Delta _{2}=-\omega _{b}$ in (**f**–**j**). The other parameters are same as in Fig. 3.

Next, we plot five different distant bipartite entanglements as a function of $\Delta _{1}/\omega _{b}$ and $\Delta _{2}/\omega _{b}$ for different single photon hopping factor $\Gamma$, while keeping both the magnon detuning in resonance with blue sideband regime i.e. $\Delta _{m_{1}}=\Delta _{m_{2}}=\omega _{b}$ in Fig. 3. For $\Gamma =0.5\omega _{b}$, the quantity $E_{c_{1}-c_{2}}^{N}$ attains optimal value around i.e. $\Delta _{1}=\Delta _{2} =-0.5\omega _{b}$ whereas for off resonant cavities, we also get finite values of $E_{c_{1}-c_{2}}^{N}$ as shown in Fig. 3a. However, the cavity-magnon entanglements $E_{c_{1}-m_{2}}^{N}(=E_{c_{2}-m_{1}}^{N}$) become maximum for two different values of cavity detunings which are $\Delta _{1}=\Delta _{2} = 0$ and $\Delta _{1}=\Delta _{2} = -\omega _{b}$ as shown in Fig. 3b. In addition, we obtained the optimal cavity-phonon entanglements $E_{c_{1}-b_{2}}^{N}(=E_{c_{2}-b_{1}}^{N})$ at $\Delta _{1}=\Delta _{2} = -\omega _{b}$ as shown in Fig. 3c. It can be seen that if we increase the single photon hopping factor upto $\Gamma = 0.8\omega _{b}$ then the bipartite entanglement in between both the cavity modes $E_{c_{1}-c_{2}}^{N}$ becomes maximum for two cases i.e. either both the cavity detunings should be in red sideband regime ($\Delta _{1}=\Delta _{2} = -\omega _{b}$) or in blue sideband regime ($\Delta _{1}=\Delta _{2} = \omega _{b}$) as shown in Fig. 3d. In addition, in the density plots of the bipartite quantities $E_{c_{1}-m_{2}}^{N}=E_{c_{2}-m_{1}}^{N}$ the region corresponding to red sideband regime start to decrease whereas the region corresponding to resonant cavities increases as shown in Fig. 3e. Moreover, the quantities $E_{c_{1}-b_{2}}^{N}=E_{c_{2}-b_{1}}^{N}$ show the finite values for a broad range of cavity detunings and attain maximum value for $\Delta _{1}=\Delta _{2} = -1.5\omega _{b}$ as shown in Fig. 3f. On further increasing the value of $\Gamma$ and keeping it at $\Gamma = \omega _{b}$, the quantity $E_{c_{1}-c_{2}}^{N}$ again becomes maximum for two cases i.e. for $\Delta _{1}=\Delta _{2} = -0.5\omega _{b}$ and $\Delta _{1}=\Delta _{2} = -1.5\omega _{b}$ as shown in Fig. 3g whereas the quantities $E_{c_{1}-m_{2}}^{N}=E_{c_{2}-m_{1}}^{N}$ attain maximum value only when both the cavity detunings are nearly resonant with blue sideband regime as given in Fig. 3h. However, both the quantities $E_{c_{1}-b_{2}}^{N}=E_{c_{2}-b_{1}}^{N}$ attain maximum value only for very far off-resonant cavities $\Delta _{1}=\Delta _{2} = -2\omega _{b}$ whereas for a broad range of negative cavity detunings both these distant entanglements almost become negligible however for a positive value of $\Delta _{1}/\omega _{b}$ and $\Delta _{2}/\omega _{b}$ both the bipartite entanglements attain finite values $E_{c_{1}-b_{2}}^{N}=E_{c_{2}-b_{1}}^{N}$ as shown in Fig. 3i. Overall, it can be also seen from Fig. 3 that as the cavity-cavity photon hopping strength $\Gamma$ continue to increase, concentration of various bipartite entanglements in density plots decrease significantly. This is because within a certain range, the photon hopping strength is positively correlated with the bipartite entanglement, but when it is increases continuously, the quantum system will undergo degradation, leading to a decrease in bipartite entanglement.

**Figure 5:**
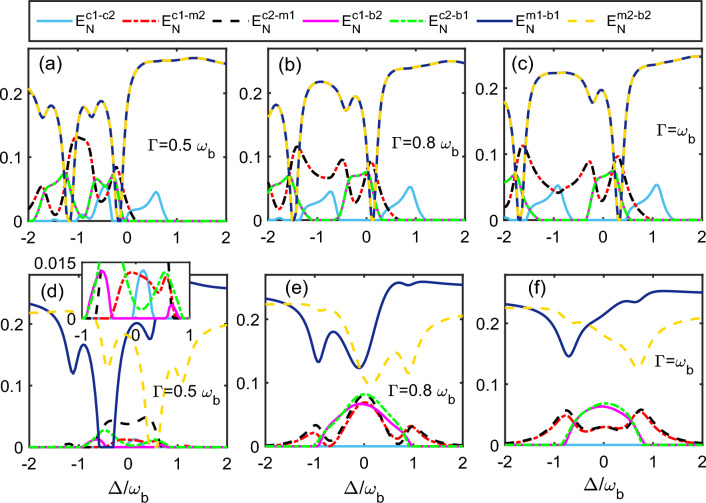
Plot of different bipartite entanglement versus $\Delta /\omega _{b}$ by taking $\Delta =\Delta _{1}=\Delta _{2}=\omega _{b}$ for the upper panel and $\Delta =\Delta _{1}=-\Delta _{2}=\omega _{b}$ for the lower panel. The other parameters are same as in Fig. 3.

Now, we study the effects of varying photon hopping factor $\Gamma /\kappa _{c}$ and normalised first cavity detuning $\Delta _{1}/\omega _{b}$ on these five bipartite entanglements. It is important to mention here that we have taken two cases: symmetric case ($\Delta _{1}=\Delta _{2}=\omega _{b}$), which correspond to upper panel and non symmetric case ($\Delta _{1}=-\Delta _{2}= \omega _{b}$) corresponding to lower panel while keeping second cavity detuning $\Delta _{2}/\omega _{b}$ fixed in Fig. 4. It can be seen that for $\Delta _{2}= \omega _{b}$, i.e. when second cavity detuning is resonant with blue sideband regime, the quantity $E_{c_{1}-c_{2}}^{N}$ becomes maximum for $\Delta _{1}$ varying in the range of $0- (-0.5\omega _{b})$ whereas photon hopping factor varies upto $0-5$ although after this range $E_{c_{1}-b_{2}}^{N}=E_{c_{2}-b_{1}}^{N}$ get finite values for both positive and negative $\Delta _{1}/\omega _{b}$ with varying $\Gamma /\kappa _{c}$ as shown in Fig. 4a. Similarly, both the bipartite quantities $E^{N}_{c_{1}-m_{2}}$ and $E^{N}_{c_{2}-m_{1}}$ get maximum for $\Delta _{1}\approx -\omega _{b}$ and after this both attain finite values again on varying $\Delta _{1}/\omega _{b}$ and $\Gamma /\kappa _{c}$ as shown in Fig. 4b and c. Moreover, the other two quantities $E_{c_{1}-b_{2}}^{N}$ and $E_{c_{2}-b_{1}}^{N}$ attain their maximum value for $\Delta _{1}/\omega _{b}$ varying in the range of $(-1)$ to $(-2)$ even for a very high value of $\Gamma /\kappa _{c}$ as shown in Fig. 4d and e. In another scenario for $\Delta _{2}= -\omega _{b}$ i.e. when second cavity detuning is resonant with red sideband regime, the quantity $E_{c_{1}-c_{2}}^{N}$ becomes maximum nearby to $\Delta _{1}/\omega _{b} = 0$ and $\Gamma \approx \kappa _{c}$ however after this it decreases very rapidly on gradually increasing $\Delta _{1}/\omega _{b}$ as well as $\Gamma /\kappa _{c}$ as shown in Fig. 4(f). For this value of second cavity detuning, it can be seen that both the bipartite entanglements $E^{N}_{c_{1}-m_{2}}$ as well as $E^{N}_{c_{2}-m_{1}}$ get maximum only around $\Delta _{1}/\omega _{b} = 0$ and $\Gamma /\kappa _{c}$ varies in between $7-10$, afterwards both these entanglements vanish as shown in Fig. 4g and h. However for this range of $\Delta _{1}/\omega _{b}$, $E_{c_{1}-b_{2}}^{N}$ and $E_{c_{2}-b_{1}}^{N}$ both become maximum for single photon hopping factor $\Gamma /\kappa _{c}$ varying in between the range of 5–7 and then both the bipartite entanglements become zero although a further increase in $\Gamma /\kappa _{c}$ give maximum values of $E_{c_{1}-b_{2}}^{N}$ and $E_{c_{2}-b_{1}}^{N}$ as depicted in Fig. 4i and j. Therefore, it can be seen that the density plots of various bipartition show completely different results depending upon that weather the second cavity detuning is resonant with blue or red sideband regime of the phonons due to deformation of YIG Sphere. This is because in case of symmetric cavities i.e. ($\Delta _{1}=\Delta _{2}=\omega _{b}$) we have only anti-stokes process whereas in case of ($\Delta _{1}=-\Delta _{2}=\omega _{b}$) both anti-stokes and stokes processes came into scenario.

**Figure 6:**
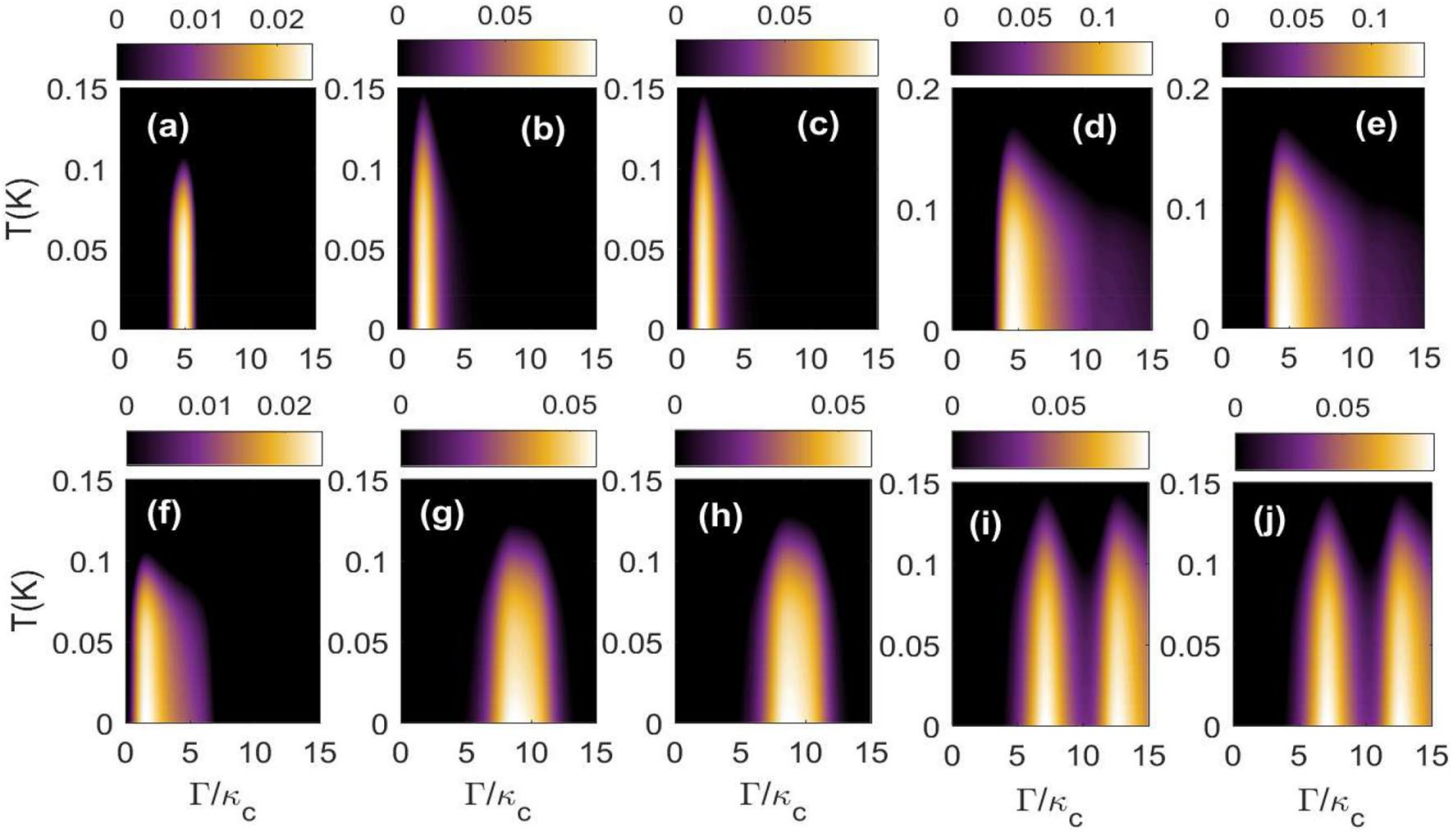
Density plot of bipartite entanglement in (**a**,**f**) $E^{N}_{c_{1}-c_{2}}$; in (**b**,**g**) $E^{N}_{c_{1}-m_{2}}$; in (**c**,**h**) $E^{N}_{c_{2}-m_{1}}$; in (**d**,**i**) $E^{N}_{c_{1}-b_{2}}$ and (**e**,**j**) $E^{N}_{c_{2}-b_{1}}$ versus temperature *T* for $\Delta _{1}=\Delta _{2}=\omega _{b}$ in (**a**–**e**) and for $\Delta _{1}= -\Delta _{2}=\omega _{b}$ in (**f**–**j**). The other parameters are same as in Fig. 3.

In Fig. 5 we plot different distant bipartite entanglements as a function of $\Delta /\omega _{b}$ for symmetric cavities where we take $\Delta =\Delta _{1} =\Delta _{2} = \omega _{b}$ (upper panel) and for antisymmetric cavities $\Delta =\Delta _{1} =-\Delta _{2} = \omega _{b}$ (lower panel). For $\Gamma = 0.5\omega _{b}$ and symmetric cavities, the bipartite quantity $E_{c_{1}-c_{2}}^{N}$ varies from 0 to 0.6  on gradually changing the normalised detuning $\Delta /\omega _{b}$ in between -1 to 1 and after this range the quantity $E_{c_{1}-c_{2}}^{N}$ becomes zero as shown in Fig. 5a. Furthermore, both the bipartite quantities $E_{m_{2}-b_{2}}^{N}$ $(E_{m_{1}-b_{1}}^{N})$ varies from 0 to 0.2 for negative values of $\Delta /\omega _{b}$ whereas for $\Delta /\omega _{b}$ greater than zero both of these quantities get saturated to a finite positive value. In addition, the bipartite quantities $E_{c_{1}-b_{2}}^{N}$ ($E_{c_{2}-b_{1}}^{N}$) as well as $E_{c_{1}-m_{2}}^{N}$ ($E_{c_{2}-m_{1}}^{N}$) become almost zero for positive values of $\Delta /\omega _{b}$ as shown in Fig. 5a. It can be seen that for this value of $\Gamma$ a significant amount of entanglement transfer takes place from $E_{m_{2}-b_{2}}^{N}$ $(E_{m_{1}-b_{1}}^{N})$ to $E_{c_{1}-b_{2}}^{N}$ ($E_{c_{2}-b_{1}}^{N}$) and $E_{c_{1}-m_{2}}^{N}$ ($E_{c_{2}-m_{1}}^{N}$) at $\Delta /\omega _{b}$ $\approx$ -0.3 and -1.2. For $\Gamma = 0.8\omega _{b}$, the bipartite entanglement $E_{c_{1}-c_{2}}^{N}$ become finite for $\Delta /\omega _{b}$ varying in the range of (0.3)–(1.3) and (–0.5)–(–1.3) as shown in Fig. 5b. It can be also seen that the bipartite quantities $E_{m_{2}-b_{2}}^{N}$ $(E_{m_{1}-b_{1}}^{N})$ almost get around 0.2 for positive as well as negative values of $\Delta /\omega _{b}$ except for certain values of $\Delta /\omega _{b} \approx 0.2$ and $-1.5$ whereas $E_{c_{1}-m_{2}}^{N}$ ($E_{c_{2}-m_{1}}^{N}$) has finite values upto $\Delta /\omega _{b} \approx 0.5$ and $E_{c_{1}-b_{2}}^{N}$ ($E_{c_{2}-b_{1}}^{N}$) becomes zero even for negative values of $\Delta /\omega _{b}$. In this case we get maximum entanglement transfer from $E_{m_{2}-b_{2}}^{N}$ $(E_{m_{1}-b_{1}}^{N})$ to $E_{c_{1}-b_{2}}^{N}$ ($E_{c_{2}-b_{1}}^{N}$) and $E_{c_{1}-m_{2}}^{N}$ ($E_{c_{2}-m_{1}}^{N}$) around $\Delta /\omega _{b}$ $\approx$ 0.1 and -1.5. If we increase further single photon hopping factor upto $\Gamma = \omega _{b}$ then the quantity $E_{c_{1}-c_{2}}^{N}$ remains finite only for $\Delta /\omega _{b}$ varying in the range of $(0.5)-(1.5)$ as well as $(-0.5)-(-1.5)$ whereas $E_{m_{2}-b_{2}}^{N}$ $(E_{m_{1}-b_{1}}^{N})$ almost gets around 0.25 except at certain values of the $\Delta /\omega _{b}$ for which the entanglement transfer takes place in between the different bipartite correlations as shown in Fig. 5c. In this case $E_{c_{1}-m_{2}}^{N}$ ($E_{c_{2}-m_{1}}^{N}$) has finite values upto $\Delta /\omega _{b} \approx 1.0$ however $E_{c_{1}-b_{2}}^{N}$ ($E_{c_{2}-b_{1}}^{N}$) qualitatively remains the same as depicted in Fig. 5c. It can be seen that the maximum entanglement transfer from $E_{m_{2}-b_{2}}^{N}$ $(E_{m_{1}-b_{1}}^{N})$ to $E_{c_{1}-b_{2}}^{N}$ ($E_{c_{2}-b_{1}}^{N}$) and $E_{c_{1}-m_{2}}^{N}$ ($E_{c_{2}-m_{1}}^{N}$) takes place around values $\Delta /\omega _{b}$ $\approx$ 0.5 and -1.8. Now for antisymmetric cavities $\Delta =\Delta _{1} =-\Delta _{2} = \omega _{b}$ and single photon hopping factor $\Gamma = 0.5\omega _{b}$ it can be seen that both the bipartite entanglements $E_{m_{2}-b_{2}}^{N}$ $(E_{m_{1}-b_{1}}^{N})$ have finite values with a varying $\Delta /\omega _{b}$ although for few values both become zero as shown in Fig. 5d. All other bipartite entanglements have very small values for this value of $\Gamma$. For $\Gamma = 0.8\omega _{b}$ the bipartite entanglement $E_{c_{1}-c_{2}}^{N}$ becomes zero whereas the quantities $E_{m_{2}-b_{2}}^{N}$ $(E_{m_{1}-b_{1}}^{N})$ have finite values from $(0.1)-(0.25)$ as shown in Fig. 5d. Moreover, the bipartite entanglements $E_{c_{1}-b_{2}}^{N}$ ($E_{c_{2}-b_{1}}^{N}$) increases for this value of $\Gamma$ and become finite for a varying $\Delta /\omega _{b}$ in between the range of (– 1)–(1) whereas $E_{c_{1}-m_{2}}^{N}$ ($E_{c_{2}-m_{1}}^{N}$) also increases and varies from 0–0.07(0.08) with $\Delta /\omega _{b}$ as depicted in Fig. 5d. With a further increment in $\Gamma$ both the bipartite entanglements $E_{c_{1}-m_{2}}^{N}$ ($E_{c_{2}-m_{1}}^{N}$) becomes finite over whole range of varying $\Delta /\omega _{b}$ whereas all other bipartite entanglements qualitatively remain the same (like earlier case of $\Gamma = 0.8\omega _{b}$ ) as shown in Fig. 5f. It can be also seen that in case of symmetric microwave cavities when both the cavity detunings are kept fixed at blue sideband regime and only anti stokes process dominates, we have finite cavity-cavity entanglement whereas for antisymmetric cavities we get both stokes and anti stokes processes and hence it almost gives negligible cavity-cavity entanglement for any value of single photon hopping strength $\Gamma$. In addition, for symmetric cavities we also get maximum entanglement transfer from directly coupled modes (magnon-phonon) to indirectly coupled modes (cavity-magnon) as shown in Fig. 5.

We study the density plots of different distant bipartite entanglements as a function of environmental temperature *T* and single photon hopping factor $\Gamma /\kappa _{c}$ for symmetric case i.e. $\Delta _{1}=\Delta _{2}=\omega _{b}$ (upper panel) and non symmetric case i.e. $\Delta _{1}=-\Delta _{2} = \omega _{b}$ (lower panel). For second cavity detuning $\Delta _{2} =\omega _{b}$ it can be seen that for $T\sim 0.1K-0.15K$, all the three bipartite entanglements $E^{N}_{c_{1}-c_{2}}$, $E^{N}_{c_{1}-m_{2}}$ and $E^{N}_{c_{2}-m_{1}}$ are finite for a very narrow range of $\Gamma /\kappa _{c}$ as shown in Fig. 6a–c whereas the other two bipartitions $E^{N}_{c_{1}-b_{2}}$ and $E^{N}_{c_{2}-b_{1}}$ remain finite for $\Gamma /\kappa _{c}$ varying in between $5-10$ as depicted in Fig. 6d–e. For second cavity detuning $\Delta _{2} = -\omega _{b}$ (lower panel), three bipartitions $E^{N}_{c_{1}-c_{2}}$, $E^{N}_{c_{1}-m_{2}}$ and $E^{N}_{c_{2}-m_{1}}$ remain finite for a wider range of varying $\Gamma /\kappa _{c}$ as shown in Fig. 6f– whereas the other two correlations $E^{N}_{c_{1}-b_{2}}$ and $E^{N}_{c_{2}-b_{1}}$ become maximum in two different regions of varying $\Gamma /\kappa _{c}$ as shown in Fig. 6i and j. Moreover, in case of antisymmetric cavities, on gradually increasing the environmental temperature *T* all the five distant bipartitions show finite value of entanglement for a broader range of single photon hopping strength $\Gamma$ as compared to symmetric cavities. Hence as compared to results discussed in Fig. 5 here antisymmetric cavities are showing enhanced concentration of the bipartite entanglement with environmental temperature *T*.

## Conclusion

We present an experimentally feasible scheme based on coupled magnomechanical system where two microwave cavities are coupled through single photon hopping parameter $\Gamma$ and each cavity also contains a magnon mode and phonon mode. We have investigated continuous variable entanglement between distant bipartitions for an appropriate set of both cavities and magnons detuning and their decay rates. Hence, it can be seen that bipartite entanglement between indirectly coupled systems are substantial in our proposed scheme. Moreover, in our present scheme cavity-cavity coupling strength also plays a key role in the degree of bipartite entanglement and its transfer among different direct and indirect modes. This scheme may prove to be significant for processing continuous variable quantum information in quantum memory protocols.

## Data Availability

The corresponding author will provide the datasets used and/or analyzed during the current work upon reasonable request.
